# Morpho-physiological parameters, nutritional status and water use efficiency of Zebda mango in relation to biochar and hydrogel application under semi-arid region

**DOI:** 10.1038/s41598-025-26304-6

**Published:** 2025-11-21

**Authors:** Ayman E. Shaban, Hossam M. Moawad, Mahmoud M. Abd El-Migeed, Nagah E. Ashour, Ahmed A. Rashedy

**Affiliations:** 1 Pomology Department, Faculty of Agriculture, Cairo University, Giza, Egypt; 2 Pomology Department, National Research Center, Dokki, Giza Egypt

**Keywords:** *Mangifera indica* L. fruit drop, TSS, WUE, Mineral content, Chlorophyll, Ecology, Ecology, Environmental sciences, Plant sciences

## Abstract

Irrigation water conservation techniques are an effective tool for maximizing irrigation water utilization, especially in coarse sandy soils under semi-arid conditions. Therefore, the objective of this research was to investigate the impacts of soil application of biochar (BC) at 7, 14 kg tree^− 1^, hydrogel (HD) at 50–100 g tree^− 1^ and without application (control) on the morpho-physiological, nutritional status and productivity of Zebda mango trees during two seasons. Results indicated that, soil application of 14 kg BC tree^− 1^, followed by 100 g HD tree^− 1^ was more effective in enhancing growth and fruit yield. BC at rate of 14 kg tree^− 1^ increased number of leaves by a percentage reached to 22.27 and 32.05%, leaves area by 18.51 and 18.51, shoot length by 21.19 and 17.95% and chlorophyll content by 81 and 51%, while it decreased leaves proline content by 9.15 and 13.78% compared to the control in the first and second seasons, respectively. Moreover, 14 kg BC increased leaf N concentration by 42.98 and 27.2%, leaf P by 75 and 47.62%, leaf K by 5% and 9.64% and leaf Mg by 27 and 6% compared to the control in the first and second seasons, respectively. Also, it improved the percentage of final fruit set by 50% and 38.09%, number of fruit by 29.47% and 22.19%, fruit weight by 20.46% and 12.76%, and increased both fruit yield and water use efficiency by 55.98 and 37.79%, while it decreased fruit drop by 2.79 and 2.16% compared to the control in the first and second seasons, respectively. Furthermore it increased the percentage of TSS by 17.09% and 18.27%, titratable acidity by 21.43% and 12.63%, ascorbic acid by 23.56% and 18%, and total sugars by 27.92% and 3.19% compared to the control in the first and second seasons, respectively.

## Introduction

Mango (*Mangifera indica* L.) is regarded as one of the oldest and preferable tropical fruits worldwide. In Egypt, the mango cultivated area was approximately 121,800 ha which produce about 1,173,000 tons^1^. Agriculture sector consumes 70% the world’s freshwater, which makes the optimization of water resource usage critical. Water scarcity is a major challenge in arid and semiarid regions, particularly under climate change, which increases the competition for resources such as water, soil and energy. Increasing population growth led to a significant gap in irrigation water demands, water resources and food security, leading to water poverty^2^. This challenge requires an effective irrigation scheduling regimes and water conservation techniques^3^.

Sandy soils in arid zones like Egypt face many constraints, like low organic matter content, limited soil holding capacity, scarce water resources, high pH and high water losses due to evapotranspiration^4^. Under these conditions, searching for soil amendments is a must to improve overall crop performance^5^. Among the most effective soil amendments are hydrogel (HD) and biochar (BC)^6^.

BC is a carbon-rich material produced from the pyrolysis of organic biomass under oxygen-limited conditions^7^. It contains high-carbon byproducts and compact aromatic structures, which effectively extended carbon retention for long years and resist their decomposition in the soil^8,9^. BC provides numerous benefits to soil, such as improved water retention, decreased heavy metal availability, stimulate soil microbial activity and minimized nutrient leaching^10–12^. Recent studies indicated that, application of BC at 2, 4, and 6 ton per feddan improved yield, fruit quality and water use efficiency (WUE) of apple fruit^13,14^. Also, BC application improved the productivity and quality of grape^15^ as well as it improved the productivity of some field crops^16^. BC has a multifunctional roles in improving soil quality such as improving water retention via porous structure. Also, it increases nutrient retention and a viability via its high cation exchange capacity. Moreover, it enhances soil biological activity and alleviate climate change impacts by carbon sequestration which stores stable carbon forms in the over extended periods, thereby restoring reclaimed degraded lands and enhancing plant production^17,18^. Application of BC at 40 Mg ha^− 1^ improved Sufaid Chaunsa mango growth, chlorophyll content, photosynthesis, fruit yield and fruit quality^5^. Furthermore, BC improve root system function and architecture which helps in water uptake and promoting nutrients^19^.

HD is regarded as a water-saving substance that can deal with water shortage. It is a high-molecular-weight that can absorb 400–1500 g of water per 1 g HD. High molecular weight that provides efficient liquid absorption^20,21^. It can support drought tolerance, increase soil water-holding capacity, delay wilting and enhance plant growth^22^. Also, it improve water conservation, yield and WUE of many fruits such as mandarin^23^, mango^6^ and olive^24^. It can serve as fertilizers carriers which enhance soil fertility and plant growth through absorbing nutrients and releasing them later^25^. There is scarce literature on the impact of BC and HD on growth and productivity of Zebda mango trees. So, the goal of this experiment was to investigate the efficiency of BC and HD on the growth, fruiting, yield, fruit quality, nutritional status and WUE of Zebda mango trees.

## Materials and methods

### Experimental details and treatments

This experiment was conducted over during two successive seasons (2020–2021 and 2021–2022) on fifteen-year-old Zebda mango orchard. The trees were cultivated at 5 × 3 m in sandy soil in Agricultural Production and Research Station, National Research Centre,, El-Behaira Governorate, Egypt (latitude 30.8667 N, and longitude 30.1667 E). Horticultural practices were applied for all trees. The meteorological data was presented in Table 1.

**Table 1:** Meteorological data (temperature. Relative humidity and wind speed) during experimental periods

Month	First Season				Second Season			
	Temperature (°C)		Relative Humidity (%)	Wind (m s ^− 1^)	Temperature (°C)		Relative Humidity (%)	Wind (m s ^− 1^)
	Minimum	Maximum			Minimum	Maximum		
	2020/2021	2020/2021	2020/2021	2020/2021	2021/2022	2021/2022	2021/2022	2021/2022
November	10.49	28.53	64.57	3.66	13.01	33.18	63.86	3.05
December	8.58	26.22	63.8	3.12	6.8	24.18	69.21	3.65
January	5.2	27.06	63.97	3.38	2.38	21.63	68.41	3.44
February	6.11	27.96	64.92	3.56	5.14	24.7	67.33	3.48
March	5.86	33	63.22	4.43	4.01	29.99	62.4	4.54
April	7.4	40.89	53.96	4.26	8.99	40.83	51.34	4.32
May	15.07	42.72	44.08	5.63	12.73	42.31	47.86	5.61
June	16.25	41.53	47.64	5.81	18.55	45.35	48.82	5.47
July	20.32	44.13	48.2	5.53	19.88	41.3	49.56	5.31
August	20.94	43.97	50.45	5.66	21.31	41.86	51.19	5.19
September	18.85	41.07	53.61	5.5	19.68	41.31	52.07	4.95
October	16.23	35.53	56.92	4.21	16.14	40.39	58.85	4.23

NASA 2025. Data resources. Cited in https://power.larc.nasa.gov/data-access-viewer/. Access in 12/08/2025.

### Soil and irrigation water analyses

Soil samples were taken from the soil at four depths (0–15 cm, 15–30 cm, 30–45 cm and 45–60 cm). Irrigation water was obtained from an irrigation channel. Soil chemical properties (Table 2) were determined at the laboratory of Soil Dept. National Research Centre, as follows: Soil pH and EC were measured in 1:2.5 (soil: water suspension) and in soil paste extract, respectively. Chemical analysis (Table 2) and physical analysis (Table 3) of the soil were analyzed.

For soil physical properties (Table 3), The Pipette method^26^ was used to determine the soil particle volume distribution. Soil moisture content at permanent wilting point (PWP) and at field capacity (FC) were measured^26^.

**Table 2:** Chemical properties of the experimental soil.

Depth (cm)	PH 1:2.5	EC dS m^− 1^	Soluble cations (mqL^− 1^)				Soluble anions (mqL^− 1^)			
			Ca^++^	Mg^++^	K^+^	Na^+^	HCO_3_^−^	CO_3_^−^	SO_4_^−^	Cl ^−^
0–15	8.30	0.35	0.50	0.42	0.23	1.05	0.11	0.00	0.82	1.27
15–30	8.20	0.36	0.51	0.43	0.24	1.04	0.13	0.00	0.86	1.23
30–45	8.30	0.34	0.55	0.41	0.23	1.05	0.12	0.00	0.85	1.27
45–60	8.40	0.73	0.57	0.43	0.25	1.06	0.17	0.00	0.86	1.28

**Table 3:** Physical properties of the experimental soil.

Depth (cm)	Particle Size distribution %				Texture Class	FC	PWP	AW	BD (g/cm³)	TP (%)
	C. Sand	F. Sand	Silt	Clay						
0–15	14.87	78.90	4.40	1.83	Sand	10.50	4.16	6.34	1.58	40.38
15–30	14.91	78.93	4.30	1.86	Sand	10.40	4.10	6.30	1.60	39.62
30–45	14.89	78.73	4.41	1.97	Sand	10.46	4.13	6.33	1.64	38.11
45–60	14.96	78.66	4.39	1.99	Sand	10.45	4.20	6.25	1.66	37.36

FC: Field capacity; PWP: Permanent wilting point; AW: Available water; B.D: Bulk density, and TP: Total Porosity

## Chemical properties of irrigation water

Chemical analysis of irrigation water was performed using the standard methods shown in Table 4. All the measured chemical parameters describe the status of the irrigation water and it can be used normally in irrigation. Chemical analysis of irrigation water (Table 4) was analyzed.

**Table 4:** Chemical properties of irrigation water.

pH	EC dsm^− 1^	Soluble cations (mqL^− 1^)					Soluble anions (mqL^− 1^)			SAR
		Ca^++^	Mg^++^	K^+^	Na^+^	HCO_3_^**−**^	CO_3_^−^	SO_4_^−^	Cl ^−^	
7.20	0.36	0.75	0.23	0.11	2.50	0.90	0.00	0.33	2.52	3.67

## Experiment layout

**Fig. 1:**
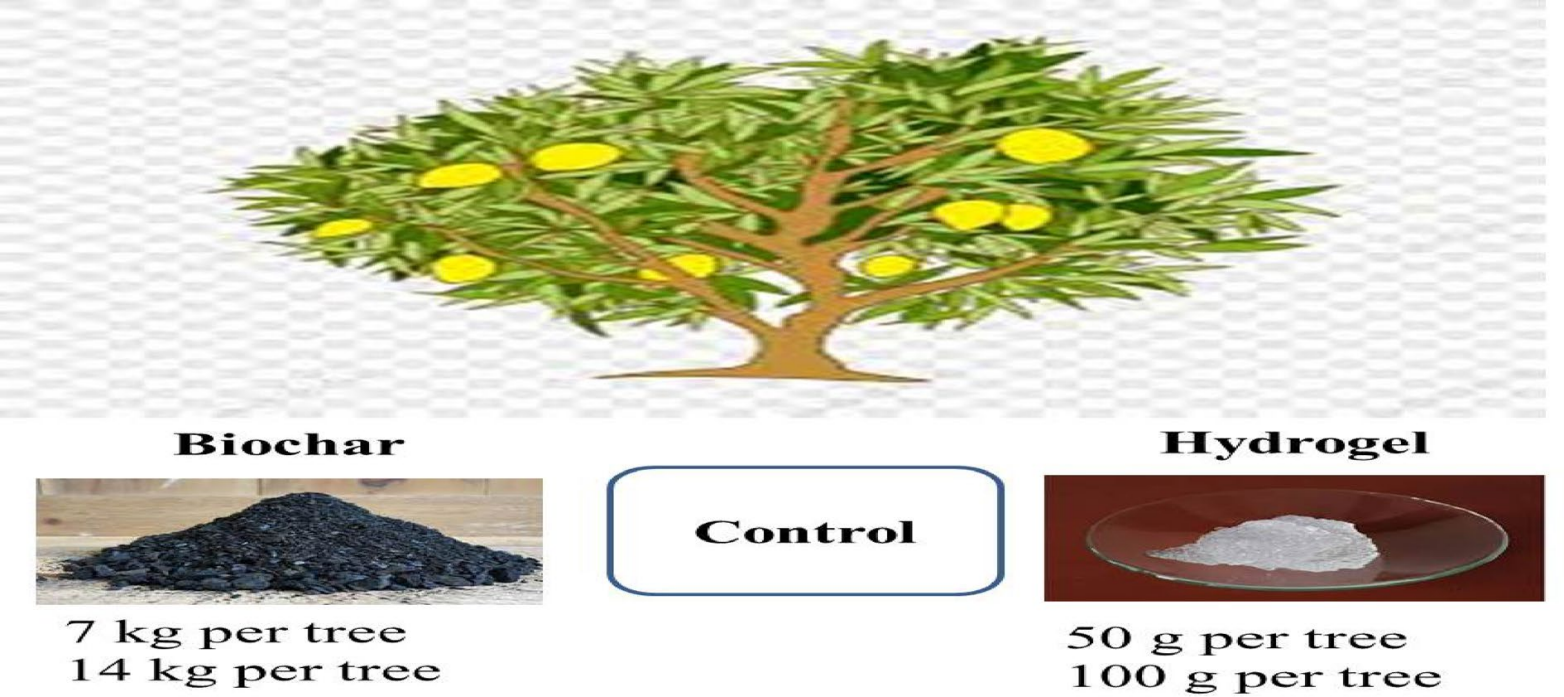
Soil application of biochar, hydrogel and control.

Forty-five trees were selected from Zebda mango cultivar for this investigation. The selected trees were arranged in five treatments with nine trees for each treatment and each three trees was treated as a replicate. Selected trees received the one of the following treatments: Control, 7 kg BC tree^− 1^, 14 kg BC tree^− 1^, 50 g HD tree^− 1^ and 100 g HD tree^− 1^ (Fig. 1). Soil amendment treatments were started in the previous November each season. The authors confirmed that all experiments were performed in accordance with relevant named institutional and/or licensing guidelines and regulations.

## Biochar description

BC is a functional material with high amount of carbon, strong adsorption capacity and rich pore structure. It was make under hypoxia conditions by pyrolysis and carbonization of biomass. Casuarina (*Casuarina equisetifolia*) tree branches were used for BC preparation, which was produced under 500 °C in an oxygen-limited environment^27^ the sample analysis was presented below (Table 5).

**Table 5:** Chemical properties of biochar.

Parameter	Value	Parameter	mg kg^− 1^	Parameter	mg kg^− 1^
Moisture %	3.18	P	23,000	Mn	87
PH	8.94	K	13,700	Zn	107
Ash %	18.58	Mg	28,300	Cu	98
EC (ds/m)	1.25	Ca	24,800	Na	10,800
C %	66.4	S	481		
N %	1.58	Fe	328		

## Hydrogel description (STOCKOSORB^®^ 500)

HD is a water-absorbent polymer with a white color and granular shape (Table 6). It was designed to improve the water holding capacity of the soil through acting as a water reservoir. It can hold water up to 700 g per 1 g HD (STOCKOSORB^®^ 660, Advance Landscape Systems, New Zealand).

**Table 6:** Chemical properties of hydrogel (STOCKOSORPO 660).

Parameter	Value	Parameter	Value
pH (1.0 g/l water)	7.7	Potassium polyacrylate, cross-linked	>=95%
Vapor pressure (20 ‘C)	< 20 hPa	Physical state	Solid
Density	0.7 g cm^− 3^	Form	Powder
Bulk density	600 kg m^− 3^	Color	White
CAS-Number	25608-12-2	Odor	Odorless

### Measurements

#### Vegetative growth parameters

In March every year, 80 branches from 4 sides of the tree were randomly chosen for measuring both vegetative and fruiting characteristics. During August, shoot length was measured (cm) of new developed flushes per shoot, shoot diameter (cm) with caliper, number of leaves were counted and leaf area (cm²) was measured (30 leaves per treatment) using the following equation^28^.

$${\text{leaf~area~}} = ~0.70~\left( {{\text{leaf~length}}~ \times ~{\text{leaf~width}}} \right)~ + ~1.06$$

#### Leaf nutritional status

Fully mature expanded leaves of similar age were collected from the previously labeled shoots on the second week of October. Whereas, 2–3 leaves from every shoot (4th and 5th leaves from the shoot base) were taken then mixed together as a composite for carrying out the following chemical analysis:

A composite leaf sample from each replicate (three trees) was oven-dried 70 º C to constant weight, and then grounded for determination the following nutrient elements as percentage of dry weight. Nitrogen using the modified micro-kjeldahl method as described by Pregl^29^. Phosphorus was estimated as described by Chapman and Pratt^30^. Potassium was determined by flame photometerically according to Brown and Lilleland^31^. Magnesium was determined as ppm were spectrophotometerically determined using atomic absorption (Model, spectronic 21 D) as described by Cottenie et al.^32^.

#### Leaf physiological parameters

It was determined in August for each season, 30 leaves per treatment were used for determination of chlorophyll concentrations using color-metrically Minolta SPAD-502 (made in Japan). Leaf water content LWC % was calculated according to Barrs^33^, from the following equation:

$${\text{LWC}}\% = ~\frac{{{\text{FW}} - {\text{DW}}}}{{{\text{FW}}}} \times 100$$

Where, FW- fresh weight; DW- dry weight. Leaf proline content (mg per g FW) was measured using the ninhydrin reaction^34^.

#### Fruiting characteristics

Initial fruit set was determined as counting number of setting fruits per panicle two weeks after petal fall for panicles on tagged shoots. Final fruit set was determined by counting number of retained fruits per panicle at harvest (first week of August). Fruit drop % was determined at harvest by calculate final fruit set from initial fruit set and final fruit set as the following:

$${\text{Initial~fruit~set\% ~ = }}\frac{{{\text{Number~of~setting~fruits}}}}{{{\text{Total~number~of~flowers}}}}{{ \times 100}}$$

$${\text{Final~fruit~set}}\% ~ = \frac{{{\text{Number~of~retained~fruits}}}}{{{\text{Total~number~of~flowers}}}} \times 100$$

$${\text{Fruit~drop~set}}\% ~ = \frac{{{\text{Initial~fruit~set~{-}~Final~fruit~set}}}}{{{\text{Initial~fruit~set~}}}} \times 100$$

#### Fruit yield and WUE

Number of fruits per tree at maturity was counted. Yield (kg tree^− 1^) was evaluated by multiplying number of fruits per tree by the average fruit weight.

Water use efficiency (kg m^− 3^) was calculated according to FAO^35^.

$${\text{WUE}} = \frac{{{\text{Yield}}\left( {{\text{kg}}} \right)}}{{{\text{Irrigation requirements (m}}^{{\text{3}}} {\text{)}}}}$$

Irrigation requirements (IR) were calculated according to Allen et al.^36^ as following equation:

$${\text{IR}} = \frac{{{\text{ETO}} \times {\text{Kc}}}}{{{\text{Ei}} - {\text{R}} + {\text{LR}}}}$$

Where Kc = crop factor^36^, Ei = irrigation efficiency (assumed 90%), R, mm rainfall and ETO = reference evapotranspiration, mm/day (estimated from the Central Laboratory for Climate - Agricultural Research Centre Egyptian Ministry of Agriculture (El-Nubaryia farm) and according to Penman-Montei; The amount of water needed for salt leaching was determined as the ratio of irrigation water salinity to drainage water salinity, or LR, mm. There was a three-day interval between irrigations. IR was 4348 and 4297 m^3^ fed^− 1^ (1 feddan = 4200 m²) in the first and second seasons, respectively.

#### Fruit physical and chemical characteristics

A sample of 15 ripe fruits from each replicate tree was taken at (August) the harvest time^37^ for determining the following fruits properties. Fruit weight (kg) was measured by digital balance with 0.0001 g sensitivity. Fruit total soluble solids (TSS) was measured in mango fruit juice using a digital refractometer^38^. The total soluble solids were expressed as a percent. Fruit titratable acidity: Mango fruit juice samples (5 ml) were used and titrated against 0.1 N sodium hydroxide in the presence of phenolphthalein as an indicator^37^. The titratable acidity was expressed as grams of citric acid percent. Ascorbic acid content was determined as mg of ascorbic acid per 100 g Juice with using 2,6-dichloro-phenol indophenol^39^. Total sugars (%) was determined according to Miller^40^. Which quantified by the 3,5-dinitrosalicylic acid method.

### Statistical analysis

The obtained data during both 2021 and 2022 experimental seasons were conducted using a randomized complete block design with one factor; each treatment was replicated three times with one tree per replicate. Data from the analytical determinations were subjected to analysis of variance (ANOVA) according to Snedecor and Cochran^41^ using COSTAT program. The least significant ranges (LSR) were used to compare between means of treatments according to Duncan^42^, at probability of 5%.

## Results and discussion

### Vegetative growth

**Fig. 2:**
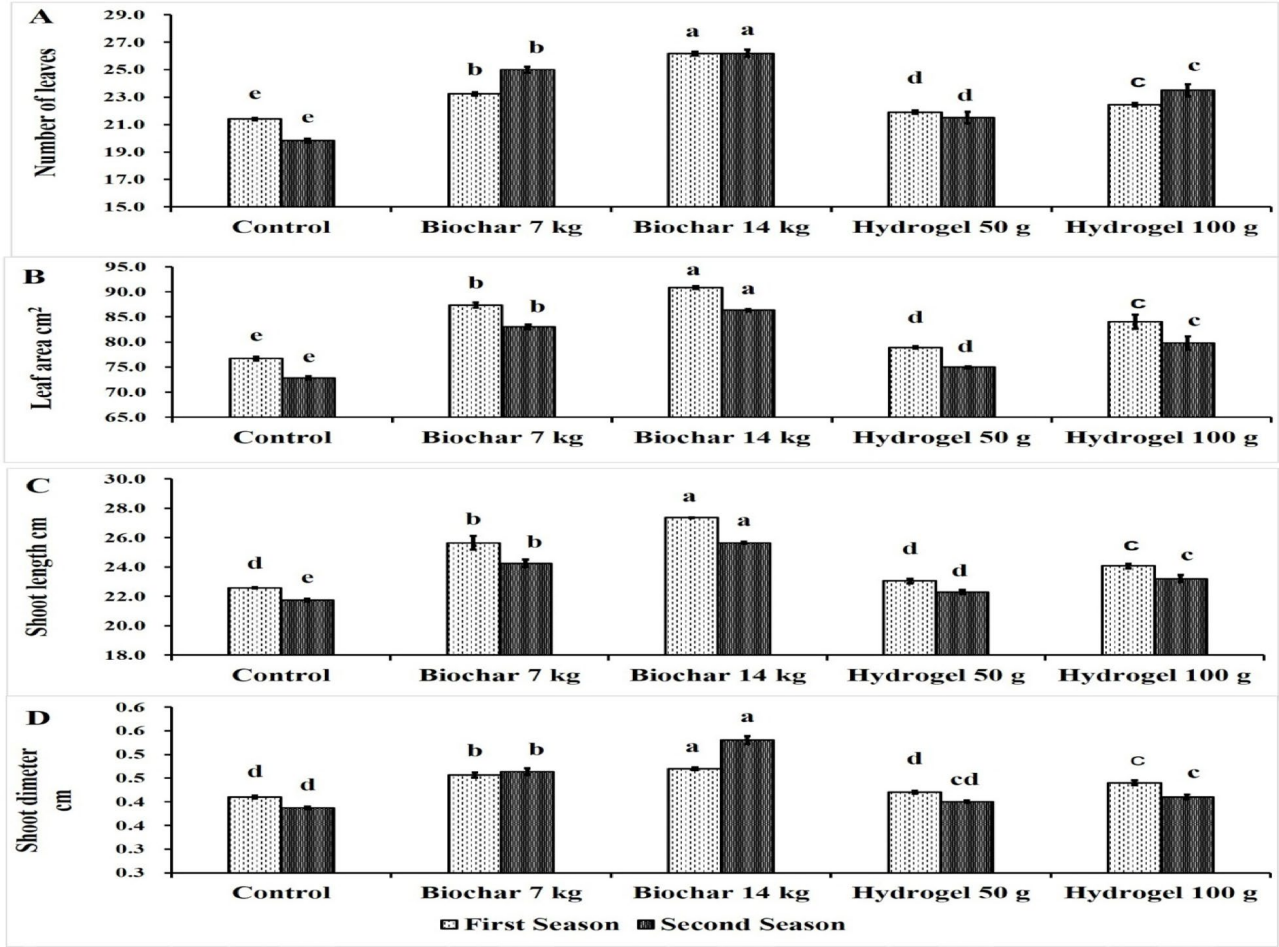
Effect of soil application of biochar (7 and 14 kg tree^− 1^) or hydrogel (50–100 g tree^− 1^) or without soil application (control), on number of leaves (A), leaf area (B), shoot length (C) and shoot diameter (D) of Zebda mango tree through two seasons. Data with similar letters are not significantly different. The means (*n* = 3 ± SE) of the data were displayed.

The data presented in Fig. 2A demonstrate the impact of BC and HD as soil amendments on the number of leaves of Zebda mango trees through two seasons. All treatments significantly improved number of leaves compared to the control in both seasons. Trees treated with BC at rate of 14 kg tree^− 1^ recorded the highest number of leaves (26.17 & 26.19), followed by BC at 7 kg tree^− 1^ (23.23 & 25), then HD at 100 g tree^− 1^ (22.45 & 23.50), then HD at 50 g tree^− 1^ (21.90 & 21.50). While the control treatment recorded the lowest number of leaves (21.40 & 19.83) in both seasons.

Figure 2B illustrates the impact of soil application of BC and HD as soil amendments on the leaf area of Zebda mango trees over two seasons. It was clear that, all treatments significantly increased leaf area compared to the control in both seasons. Also, BC at rate of 14 kg tree^− 1^ gave the highest significant leaf area (90.89 & 86.34 cm^2^) followed by BC at 7 kg tree^− 1^ (87.38 & 83.01 cm^2^), then HD at 100 g tree^− 1^ (84.02 & 79.82 cm^2^) and then HD at 50 g tree^− 1^ (78.092 & 74.98 cm^2^). While the control treatment recorded the lowest leaf area (76.69 & 72.86 cm^2^).

The presented data in Fig. 2C illustrates the impact of soil application of BC and HD as soil amendments on shoot length of Zebda mango trees through two seasons. It can be observed that, all treatments increased shoot length compared to the control with a significant value in the second season. In this concern, the highest significant shoot length was recorded by BC rate of 14 kg tree^− 1^ (27.38 & 25.65 cm) followed by 7 kg tree^− 1^ (25.65 & 24.25 cm) then HD at 100 g tree^− 1^ (24.07 & 23.21 cm) in both seasons. The lowest shoot length was recorded by control treatment (22.59 & 21.75 cm).

The presented data in Fig. 2D indicated the impact of BC and HD as soil amendments on shoot diameter of Zebda mango trees through two seasons. All treatments significantly increased shoot diameter compared to the control except for HD at 50 g in both studied seasons. Moreover, BC at rate of 14 kg tree^− 1^ gave the highest shoot diameter (0.47 & 0.53 cm) as well as BC at 7 kg tree^− 1^ (0.46 & 0.46 cm). On the contrary, control treatment produced the lowest value of shoot diameter (0.41 & 0.39 cm).

Generally, BC at rate of 14 kg tree^− 1^ succeeded in increasing number of leaves by a percentage reached to 22.27% and 32.05%, leaves area by a percentage reached 18.51% and 18.51%, shoot length by a percentage reached 21.19% and 17.95% and shoot diameter by a percentage reached 14.63% and 37.07% compared to the control in the first and second seasons, respectively. Soil amendments were effective in improving the growth of Zebda mango trees. This enhancement was furthered by increasing the amount of soil amendments. BC has previously been found to plays a vital role in enhancing tree growth, with significantly higher levels of CAT and GST found in root content under 0.5% and 1% BC treatments^10^. Also, Murtaza et al.^11^ found that, soil application of 3% BC for tomato plants improve vegetative growth of tomato. Also, the application of BC greatly improved both root development and plant growth under drought conditions^12^. This could be due to BC improved porosity and declined soil dry density which could limit anaerobic root respiration and improved root activity^43^ and mainly via enhanced antioxidant enzyme system^44,45^. Also, HD application improved vegetative growth. This could be a result of improving soil water and nutrient uptake through the application of HD. When plants require nutrients for growth, they release the nutrients that HDs have absorbed from the soil in an exchange relationship^46,47^. Increasing shoot length with BC may be due to increasing P availability and root growth^48^. Also, increasing leaf area, leaf number due to improve soil water permeability and facility root penetration and root growth^49^. Vermicompost and pistachio BC increased shoot growth of eggplant^50^.

### Leaf physiological parameters

**Fig. 3:**
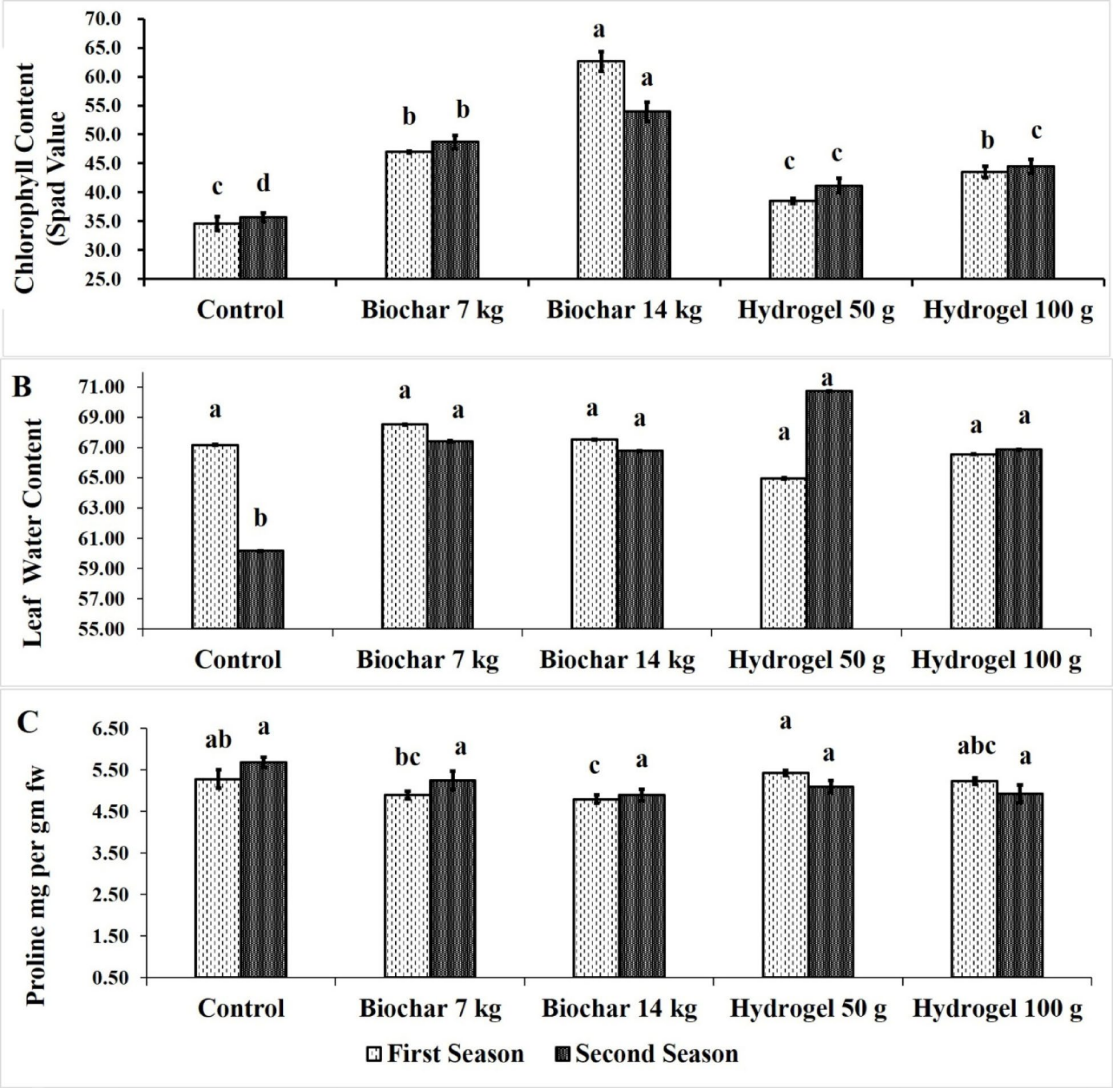
Effect of soil application of biochar (7 and 14 kg tree^− 1^) or hydrogel (50–100 g tree^− 1^) or without soil application (control), on chlorophyll content (**A**), leaf water content (**B**) and proline leaf content (**C**) of Zebda mango tree through two seasons. Data with similar letters are not significantly different. The means (*n* = 3 ± SE) of the data were displayed.

Figure 3A illustrates the impact of BC and HD as soil amendments on leaves chlorophyll content of Zebda mango trees over two seasons. All treatments improved leaves chlorophyll content compared to the control. Soil application of BC at rate of 14 kg tree^− 1^ significantly increased leaves chlorophyll content (62.63 & 53.93 Spad) followed by BC at 7 kg tree^− 1^ (46.96 & 48.70 Spad) then HD at 100 g tree^− 1^ (43.50 & 44.53 Spad) in both seasons. In contrast, the lowest leaves chlorophyll content was recorded by control treatment (34.60 & 35.70 Spad).

Figure 3B illustrates the impact of BC and HD as soil amendments on leaves water content of Zebda mango trees over two seasons. All treatments improved leaves water content compared to the control. Soil application of BC at 7 kg tree^− 1^ (68.5% & 67.4%) significantly increased leaves water content followed by BC at rate of 14 kg tree^− 1^ (67.5% & 66.7%). Also HD at 100 g tree^− 1^ increased leaves water content (66.5% & 66.8%) in both seasons. Conversely, the lowest significant leaves water content was recorded by control treatment in the second season (60.15%).

Figure 3C demonstrates the impact of BC and HD as soil amendments on leaves proline content in Zebda mango leaves over two seasons. The results showed that all treatments did not significantly increased proline content in the second season. While, BC at rate of 14 kg tree^− 1^ had the lowest significant proline content compared to the all other treatments in the first season (4.80 mg g ^− 1^ FW). While both HD at 50 g tree^− 1^ (5.43 mg g ^− 1^ FW) and control (5.28 mg g ^− 1^ FW) recorded the highest value in the first season.

Generally, biochar at rate of 14 kg tree^− 1^ increased chlorophyll content by a percentage reached to 81% and 51%, leaves water content by 0.5% and 10% and decreased leaves proline content by 9.15% and 13.78% compared to the control in both seasons, respectively. These results were in agreement with Murtaza et al.^11^ who found that, application of 3% BC for tomato plants improve rates of transpiration, photosynthesis, leaf relative water content and leaf gas exchange attributes. Application of 300 g BC increased chlorophyll content of melon plants^51^. Increasing chlorophyll content may be due to improve Mg and N uptake which necessary for chlorophyll composition^52,53^. BC increased leaf photosynthesis and chlorophyll content in many plant species such as soybean^54^ and tomato^55^.

The findings showed that, BC and HD applications successfully enhanced chlorophyll levels. Seleiman et al.^56^ observed an increase in chlorophyll levels in sunflower plants treated with BC. This improvement in chlorophyll levels could be a result of BC increasing plant growth and physiological functions like stomatal conductance, POX, and CAT activities during water scarcity^56^. Also, plants benefit from improved hydration and improved ability to open stomata with higher water availability^57^. Also, HD enhanced chlorophyll content may be due to enhancing soil moisture and nutrient uptake through the HD application. Plants uptake the nutrients absorbed by HDs from the soil in an exchange relationship when they require nutrients for growth^46,47^. Application of BC decreased proline content of Zebda mango leaves under 70% irrigation requirements^6^. These results are in harmony with those reported by Murtaza et al.^11^ who found that, BC (3%) reduced proline levels in tomato leaves. Which may be due to the increase in gas exchange and relative water content, along with the decline in proline content, resulted from the growing water availability in the soil which decreased osmotic pressure and improves the plant water uptake capacity^58^. Moreover, 3% Biochar (w/w) increased vegetative growth of tomato plants and for leaf pigment contents^11^. Both HD and BC under sandy soil with low organic matter (Table 3) maintain high soil moisture which protecting roots from relatively dry conditions during hot daytime weather when transpiration increases with increased evaporation (Table 1), making soil moisture more favorable than control plants.

### Leaf nutritional status

**Fig. 4:**
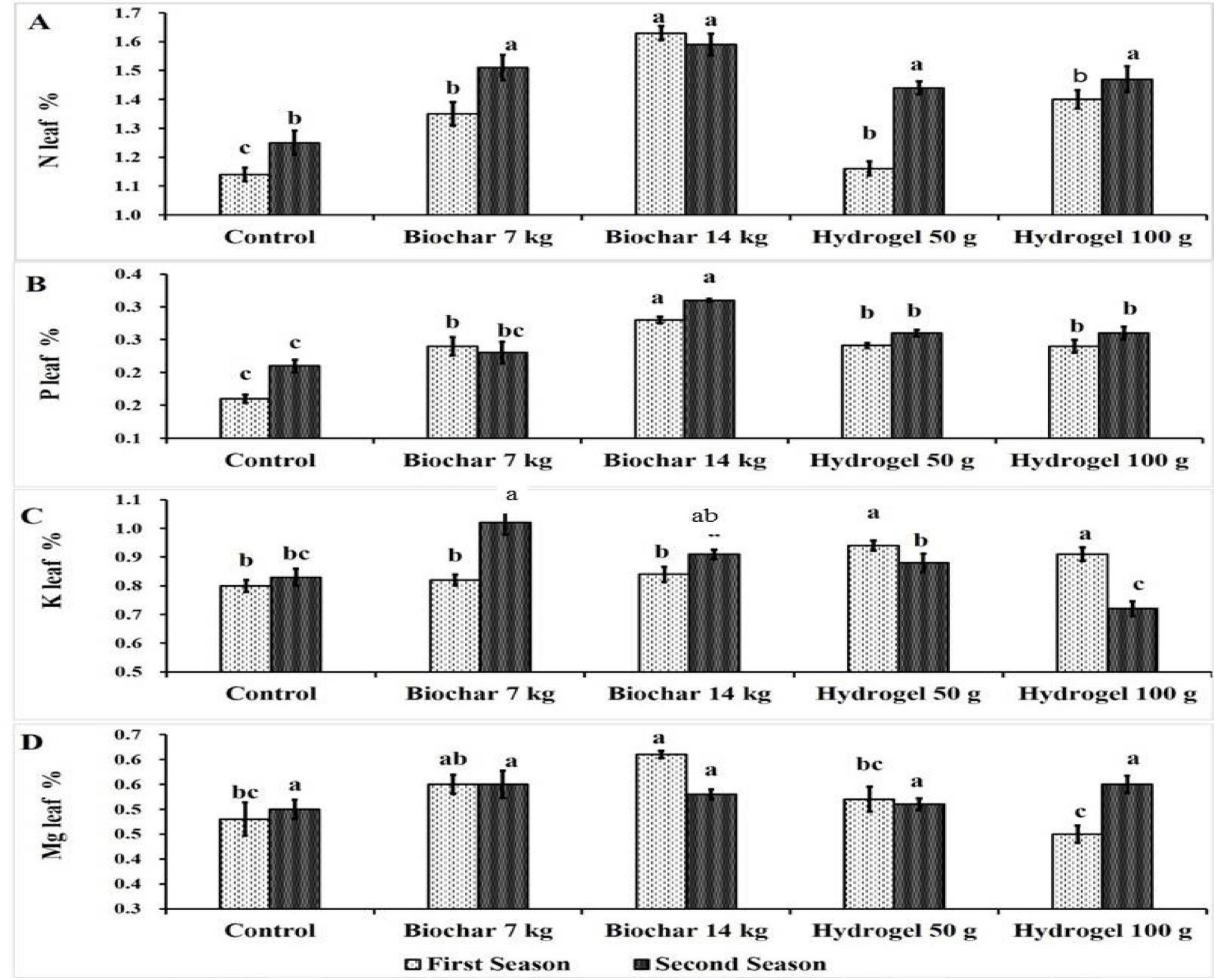
Effect of soil application of biochar (7 and 14 kg tree^− 1^) or hydrogel (50–100 g tree^− 1^) or without soil application (control), on leaf N% (**A**), leaf P% (**B**), leaf K% (**C**) and leaf Mg% (**D**) of Zebda mango tree through two seasons. Data with similar letters are not significantly different. The means (*n* = 3 ± SE) of the data were displayed.

The data in Fig. 4A indicated the impact of BC and HD as soil amendments on N leaf % through two growing seasons. The data illustrated that, BC at rate of 14 kg tree^− 1^ recorded the highest N leaf content (1.63 & 1.59%) followed by BC at 7 kg tree^− 1^ (1.35 & 1.51%) then HD at 100 g tree^− 1^ (1.40 & 1.47%), while there is no significant differences between them in the second season. In contrast, the control treatment recorded the lowest N leaf content in both seasons (1.14 & 1.25%).

Figure 4B show the impact of BC and HD as soil amendments on P leaf % of mango leaves through the two seasons. BC and HD applications significantly increased P leaf content compared to the control except for BC at 7 kg in the second season. Moreover, BC at rate of 14 kg tree^− 1^ (0.28% & 0.31%) gave the highest significant P leaf content followed by HD at 100 g (0.24% & 0.26%) and 50 g tree^− 1^ (0.24% & 0.26%) in both seasons. In contrast, the control treatment resulted in the lowest P leaf content (0.16% & 0.21%).

Figure 4C indicated the impact of BC and HD as soil amendments on K leaf % through two seasons. The data showed that, HD at 50 g (0.94 & 0.88%) and 100 g tree^− 1^ (0.91 & 0.72%) gave the highest K leaf content in the first season, while BC at 7 kg (0.82 & 1.02%) and rate of 14 kg tree^− 1^ (0.84 & 0.91%) gave the highest significant K leaf content in the second season. Conversely, the lowest value of K content was recorded by HD at 100 g tree^− 1^ (0.72%) in the second seasons and control treatment in the first season (0.83%).

Figure 4D indicated the impact of BC and HD as soil amendments on Mg leaf % of Zebda mango leaves through two seasons. The data illustrated that BC at rate of 14 kg tree^− 1^ (0.61% & 0.53%) gave the highest value of Mg content followed by BC at 7 kg tree^− 1^ (0.55% & 0.55%) and HD at 100 g tree^− 1^ (0.45% & 0.55%) in the first season. While there is no significant differences between treatments in the second seasons. Conversely, the lowest Mg content was recorded by control treatment (0.48%) and HD at 100 g tree^− 1^ (0.45%) in the first seasons.

BC and HD had no significant effect on leaf Mg % in the second season, while in the first season BC had a significant increase. Increasing Mg % due to BC and HD is little mentioned in the most previous literature^4,9,50^ except for studies on melon plants^51^. In this study Mg content in irrigation water (Table 4) and soil (Table 2) is good which make plants do not had a different between treatment since plant need to low Mg content compared to N and K. Moreover, K had antagonistic effect on Mg absorption and transition into plant^59^.

Generally, soil application of BC at rate of 14 kg tree^− 1^ increased N by 42.98% and 27.2%, P by 75% and 47.62%, K by 5% and 9.64% and Mg by 27% and 6% compared to the control in the first and second seasons, respectively. The impact of water conservations (BC and HD) on the leaf mineral content come from the direct role on the soil mineral availability and the mineral content of BC. Since, application of BC at 5 kg tree^− 1^ increased soil organic matter, K, Ca, Mn, and P^9^. Also, BC improved soil physical and chemical properties via increasing water-holding capacity, soil nutrient and microbial activities^17^. HD increase mango leaf mineral content through serves as fertilizers carriers which enhance soil fertility and plant growth through absorbing nutrients and releasing them later^25^. Moreover, BC increased root dry weight and root morphological traits and total chlorophyll^43^. Application of BC at 500 g m^− 2^ increased leaf N, P, K, Fe and Mn content of eggplant^50^. BC application with N fertilizer stimulate microbial and enzymatic activity which increased soil N content^66^. Also, water conservation materials (BC and HD) prevent mineral leaching from soil. These results were in line with Aly et al.^60^ who reported that HD polyvinylalcohol at 0.2% recorded the highest peanut yield and macronutrients (N, P, K) under 25% of available soil moisture. This may be due to absorption of N, P and K decreased under drought conditions^61^, while soil amendments like BC and HD increased soil water holding capacity and alleviate a relative reduction in soil moisture during hot weather conditions. Also, N, P, and K uptake was increased by using BC in fennel plants^62^. BC with active molecules of organic material absorb and use with the help of soil microbes which improve nutrient accumulation^63,64^. BC application increased N use efficiency^65^. Decomposition of organic matter such as BC releases CO_2_ which decrease soil pH which enhance P availability^66^. Also, BC supports ammonium and nitrate retention^67^. Moreover, BC can increase the effective K soils content^68,69^. Vermicompost and pistachio BC and 100% PWR increased content of N, P, K, Mn, Fe, of eggplant^50^. Application of 300 g BC with 100% water requirements increased plant N, K, Ca, while increasing BC rate to 450 g increased plant Mg, P^51^. Biochr at 600 mg to 1200 mg kg^− 1^ increased Malvazija Istarska grapevine photosynthetic activity and leaf N, K content^70^. Vermicompost and pistachio BC and 100% irrigation requirments increased shoot growth, WUE, higher content of N, P, K, Mn, Fe, of eggplant^50^. BC at 450 g per plant increased leaf chlorophyll content, leaf N, P, K, Ca, Mn, Mg content^51^. Movement of Ca from soil to roots decreased with decreasing soil moisture^71^.

### Fruiting characteristics

**Fig. 5:**
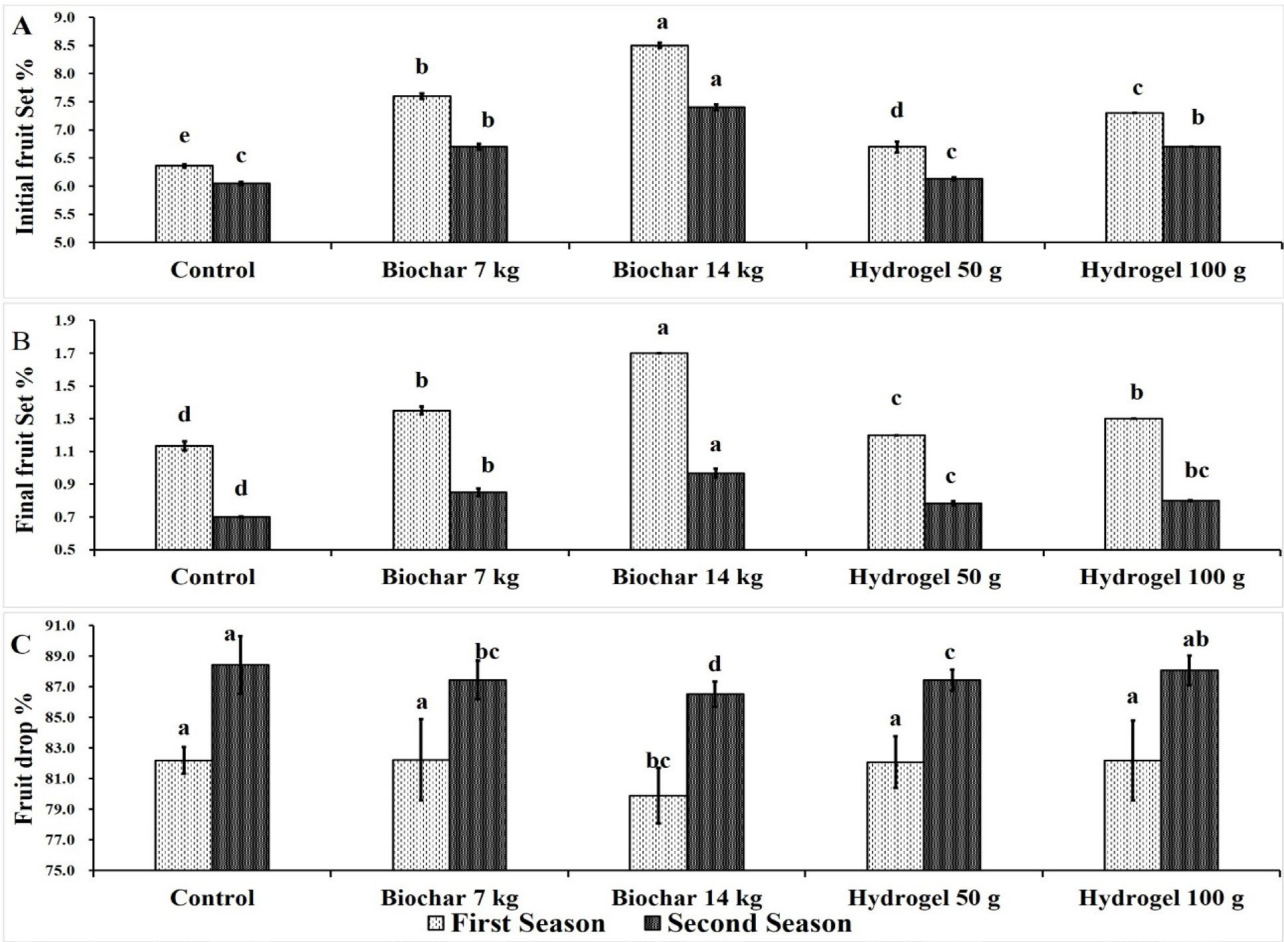
Effect of soil application of biochar (7 and 14 kg tree^− 1^) or hydrogel (50–100 g tree^− 1^) or without soil application (control), on initial fruit set% (**A**), final fruit set% (**B**) and fruit drop% (**C**) of Zebda mango tree through two seasons. Data with similar letters are not significantly different. The means (*n* = 3 ± SE) of the data were displayed.

The influence of BC and HD as soil amendments on initial fruit set of Zebda mango tree over two growing seasons was shown in Fig. 5A. First, all treatments significantly improved the initial fruit set compared to the control, except for HD at 50 g tree^− 1^. Meanwhile, the highest initial fruit set percentage was obtained by BC at rate of 14 kg tree^− 1^ (8.5% & 7.40%) followed by BC at 7 kg tree^− 1^ (6.70% & 6.13%), while the control treatment recorded the lowest initial fruit set (6.36% & 6.05%).

The impact of BC and HD as soil amendments on final fruit set% of Zebda mango trees through two seasons was presented in Fig. 5B. Generally, there is a significant difference between BC and HD compared to the control in both seasons. Moreover, BC at rate of 14 kg tree^− 1^ recorded the highest significant final fruit set (1.70 & 0.97%) followed by BC at 7 kg tree^− 1^ (1.35 &0.85%) and then HD at 100 g tree^− 1^(1.30 & 0.80%) in both seasons. In contrast, the control gave the lowest final fruit set % (1.13 &0.70%).

The effect of BC and HD as soil amendments on fruit drop% of Zebda mango trees through two seasons was presented in Fig. 5C. It can be observed that, 14 kg BC gave the lowest fruit drop (79.9 & 86.51%) compared to the other treatments. Also, in the second season this treatment followed by both HD at 50 g tree^− 1^ (87.43%) and BC 7 kg tree^− 1^ (87.44%) significantly decreased fruit drop compared to the control (88.42%).

Generally, BC at rate of 14 kg tree^− 1^ succeeded in increasing the percentage of initial fruit set by 33.64 and 22.31%, final fruit set by 50 and 38.09%, while it decreased fruit drop by 2.79 and 2.16% compared to the control in the first and second seasons, respectively. Increasing fruit set may be due to soil amendments provide more suitable soil conditions such as more water, nutrients, relative water content, chlorophyll content which improved vegetative growth and fruiting of Zebda mango trees.

In this regard, BC at 20 and 40 40 Mg/ha increased fruit retention of sufaid chaunsa mango compared to control^4^. The increasing in fruit retention due to the role of BC in improving photosynthetic efficiency, nutrient uptake and relationship and its antagonistic effect on respiration and transpiration^72^.

### Fruit yield and WUE

**Fig. 6:**
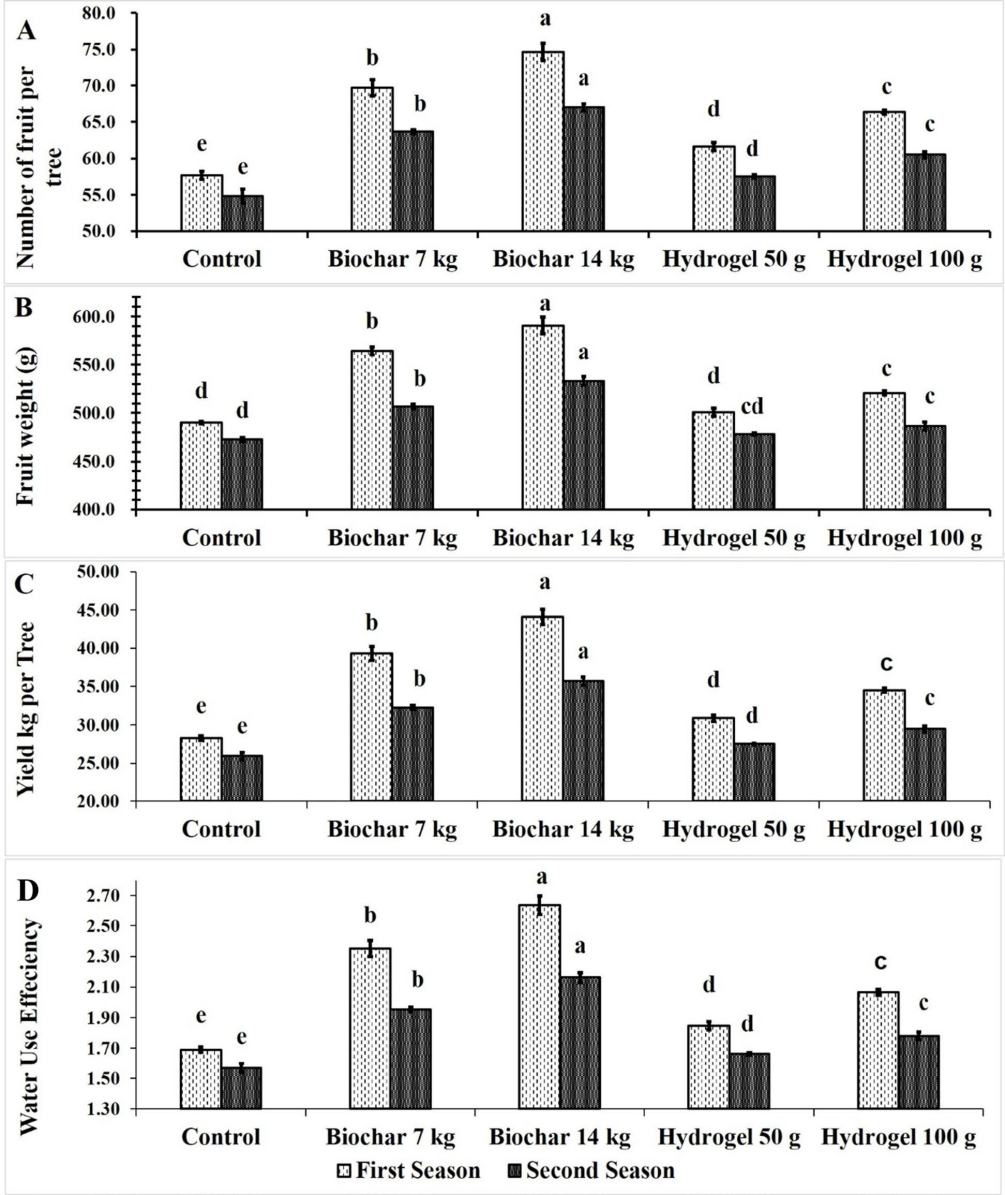
Effect of soil application of biochar (7 and 14 kg tree^− 1^) or hydrogel (50–100 g tree^− 1^) or without soil application (control) on number of fruit (**A**), fruit weight (**B**), fruit yield (**C**) and water use efficiency (**D**) of Zebda mango tree through two seasons. Data with similar letters are not significantly different. The means (*n* = 3 ± SE) of the data were displayed.

The impact of BC and HD as soil amendments on the number of mango fruits produced tree^− 1^ through two seasons was presented in Fig. 6A. BC at rate of 14 kg tree^− 1^ (74.67 & 67) followed by BC at 7 kg tree^− 1^ (69.67 & 63.67) recorded the highest significant number of fruits tree^− 1^ in both seasons. Also, HD at 100 g tree^− 1^ (66.33 & 60.50) rated statistically the third relating in increasing fruit number followed by HD at 50 g tree^− 1^ (61.67 & 57.50) then control (57.67 & 54.83). Conversely, the lowest number of fruits tree^− 1^ was obtained from the control trees.

The impact of BC and HD as soil amendments on Zebda mango fruit weight through two seasons was presented in Fig. 6B. The results showed that BC at 14 (590.7 & 533 g) and BC at 7 kg (564.3 & 506.8 g) per tree followed by HD at 100 g per tree (520.7 & 486.5 g) significantly increased fruit weight respectively in both seasons. Meanwhile, the control treatment recorded the lowest fruit weight (490.3 & 472.7 g) in both seasons, respectively. Increasing fruit weight due to using water conservation may be due to increasing vegetative growth, photosynthesis process, nutrient uptake and physiological attributes led to increasing fruit weight under both BC and HD.

The impact of BC and HD as soil amendments on Zebda mango yield (kg tree^− 1^) through two seasons was presented in Fig. 6C. BC and HD treatments significantly increased the yield compared to the control. Moreover, BC at rate of 14 kg tree^− 1^ (44.11 & 35.72 kg) gave the highest fruit yield followed by BC at 7 kg tree^− 1^ (39.33 & 32.27 kg) in both seasons then HD at 100 g tree^− 1^ (34.54 & 29.44 kg). In contrast, the control treatment produced the lowest significant fruit yield (28.28 & 25.92 kg).

Figure 6D illustrates the effects of BC and HD as soil amendments on WUE of Zebda mango trees through two growing seasons. All treatments significantly improved WUE compared to the control. In this concern, BC at rate of 14 kg tree^− 1^ (2.64 & 2.16 kg fruit m^− 3^ water) recorded the highest significant water use efficiency compared to the other treatments followed by BC at 7 kg tree^− 1^ (2.35 & 1.95 kg fruit m^− 3^ water). Also, HD at 100 g tree^− 1^ (2.07 & 1.78 kg fruit m^− 3^ water) followed by 50 g tree^− 1^ (1.85 & 1.66 kg fruit m^− 3^ water) significantly increased WUE compared to the control. While, the lowest WUE value was recorded by control (1.69 & 1.57 kg fruit m^− 3^ water) in both seasons.

Generally, soil application of BC at rate of 14 kg tree^− 1^ increased number of fruit by 29.47 & 22.19%, fruit weight by 20.46 & 12.76% and fruit yield by 55.98 & 37.79% compared to the control in both seasons, respectively. Also, BC at rate of 14 kg tree^− 1^ increased WUE by 55.98 & 37.79% compared to the control in both seasons, respectively. These results were agreement with Aly et al.^59^ who reported that, HD polyvinylalcohol at 0.2% recorded the highest peanut yield and macronutrients (N, P, K) under 25% of available soil moisture. BC increased panicum yield and nutrient content^66^. These findings may be due to BC improves soil physical properties (water retaintion, soil aggregation) and soil chemical properties (cation exchange capacity, and organic carbon) which led to resistant of nutrients for leaching^73–75^.

Fruit yield is the final product and is a cumulative effect of vegetative, physiological, soil and nutritional status of plants. Regarding the impact of soil conditioners, the findings demonstrate that both BC and HD successfully increase the number and weight of fruit. Murtaza et al.^11^ previously observed a rise in fruit production and quality attributed to the use of BC, with a 3% application leading to increased plant yield in semi-arid and arid areas. Also, BC at 6 t per feddan increased fruit weight and fruit yield of Anna apple fruit^13^. Tomato yield increased by 4%, 16%, 8%, and 3% when irrigated with varying levels of freshwater under different water deficit conditions (100% ETc, 80% ETc, 60% ETc, and 40% ETc). The effectiveness of BC might result from its capacity to maintain water, enhance porosity, and provide nutrients to plants in water stress conditions. Also increasing fruit set% and decreasing fruit drop% led to increasing fruit number which led to increase fruit yield. Increasing water holding capacity led to increase fruit weight which increase fruit yield. Also, HD increased fruit component and yield. These findings aligned with Alshallash et al.^75^ who reported that, HD led to improved productivity in mango cv. Shelly. More recently Shaban et al.^6^ found increasing fruit yield of Zebda mango fruit treated with (100 g) HD The positive effect of HD includes enhancing crop yield and quality in sandy soils by holding water and nutrient retention, leading to increased water and fertilizer use efficiency^76,77^. Moreover, it improves the root-soil environment and establishes a beneficial ecological condition for root growth^78^, leading to improved nutrient availability and increased root absorption and synthesis abilities^75,79^.

The highest WUE was recorded by 50% of available soil moisture and using polyvinylalcohol at 0.2%^59^. Using HD polyvinylalcohol at 0.2% recorded the lowest evapotranspiration in peanut crop^59^. These results may be due to HD stored the water and helped the plant to release it against drought^80,81^. BC at 3% (w/w) increased WUE of tomato plants and for leaf pigment contents^11^. WUE consider the economic environmental product of the agriculture. The application of BC and HD increased WUE in the first and second seasons respectively (Fig. 6D). BC followed by HD specially at high level of application increased greatly WUE. Since BC at 14 Kg tree^− 1^ led to improved WUE. The Improvement in WUE using 3% BC was recorded previously by Murtaza et al.^11^ who found improving WUE of tomato plants subjected to water deficit. The benefits of BC might be attributed to the ability of BC to conserve water, enhance porosity, and supply nutrients to plants in water stress conditions. In lettuce plants grown under (100%, 85%, 70% and 60% of full irrigation requirements) treated by HD at rate (0, 0.1, 0.2 and 0.3% w/w) improved WUE, by improving plant growth, soil properties, chlorophyll content, and leaf numbers. The highest water use efficiency was achieved by using HD at 0.3% concentration and supplying 85% of required irrigation, without significantly reducing yield^78^.

### Fruit physical and chemical fruit characteristics

**Fig. 7:**
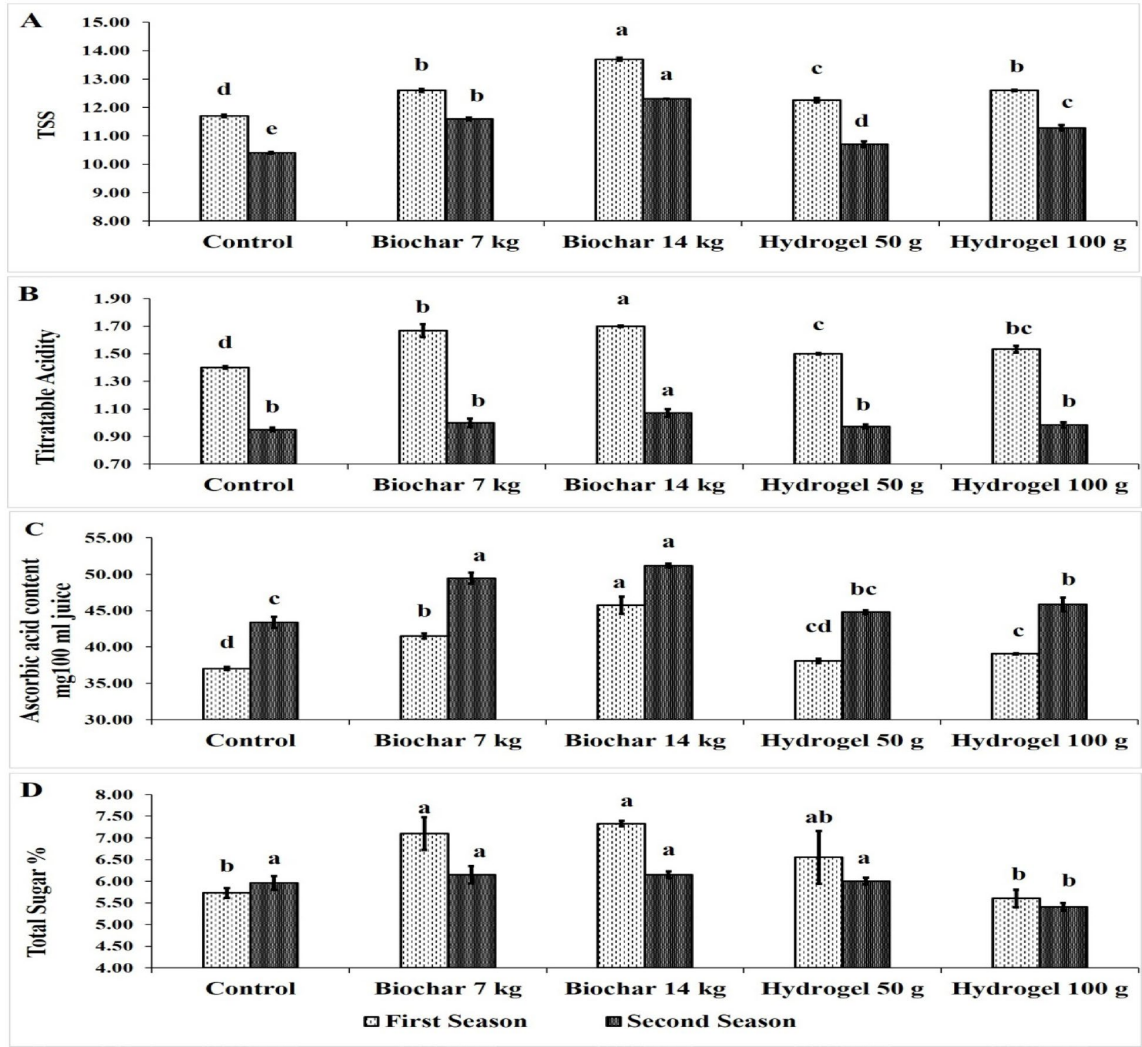
Effect of soil application of biochar (7 and 14 kg tree^− 1^) or hydrogel (50–100 g tree^− 1^) or without soil application (control), on fruit titratable acidity (**A**), ascorbic acid content (**B**) and fruit total sugars content (**C**) of Zebda mango tree through two seasons. Data with similar letters are not significantly different. The means (*n* = 3 ± SE) of the data were displayed.

The impact of BC and HD as soil amendments on TSS of Zebda mango fruit over two seasons was presented in Fig. 7A. Generally, all treatments improved TSS compared to the control. Also, trees treated with the 14 kg tree^− 1^ (13.70 & 12.30 Brix) exhibited the highest significant TSS followed by BC at 7 kg tree^− 1^ (12.60 &11.60 Brix) then HD at 100 g tree^− 1^ (12.60 & 11.28 Brix) in both seasons. On the other hand, the control treatment recorded the lowest TSS fruit content (11.70 & 10.40 Brix).

The presented data in Fig. 7B showed the effects of BC and HD as soil amendments on Zebda mango fruit acidity in both seasons. BC at rate of 14 kg tree^− 1^ (1.70 & 1.07) resulted in the highest significant fruit acidity in both seasons. Also, all treatments increased fruit acidity in the first season compared to the control treatment, whereas in the second season, only BC at 14 (1.07) recorded a significant increase than the control (0.95).

The Fig. 7C illustrates the impact of BC and HD as soil amendments on ascorbic acid content of Zebda mango fruits in both growing seasons. BC at rate of 14 kg tree^− 1^ (45.76 & 51.20 mg 100ml^− 1^) gave the highest significant ascorbic acid content of fruits than the all other treatments, followed by BC at 7 kg tree^− 1^ (41.50 & 49.46 mg 100ml^− 1^) then HD at 100 g tree^− 1^ (39.07 & 45.87 mg 100ml^− 1^). While the control treatment recorded the lowest values (37.03 & 43.38 mg 100ml^− 1^).

The Fig. 7D illustrates the effects of BC and HD as soil amendments on total sugars content of Zebda mango fruit in both seasons. BC at rate of 14 kg tree^− 1^ (7.33 &6.15%) and 7 kg tree^− 1^ (7.10 & 6.15%) recorded the highest value of fruit total sugars content with a significant value in the first season compared to the control (5.73 &5.96%) In contrast, HD at 100 g tree^− 1^ (5.60 & 5.41%) recorded the lowest fruit total sugars content in both seasons. BC at 20 and 40 Mg/ha increased fruit sugar contents of sufaid chaunsa mango compared to control^4^. This may be due to BC increased plant water and nutrients^82^ as well as increasing leaf area which produce more carbohydrates and increase fruit sugars content^83^.

Generally, soil application of 14 kg BC tree^− 1^ increased TSS by 17.09 & 18.27, titratable acidity by 21.43 &12.63%, ascorbic acid by 23.56 &18% and total sugars by 27.92 & 3.19% compared to the control in both seasons, respectively. Fruit chemical attributes TSS, acidity, ascorbic acid content and total sugars. In this regard, BC at 20 and 40 40 Mg/ha increased fruit TSS of Sufaid Chaunsa mango compared to control^4^. Also, BC at 6 t per feddan increased fruit TSS and fruit acidity of Anna apple fruit^13^. While, BC at 22 t ha^− 1^ had no significant effect on TSS of grape (cv. Merlot) parameter.

Application of BC and HD successfully increased fruit TSS, ascorbic acid content, acidity content and total sugars content. The obtained data were consistent with Alshallash et al.^76^ who reported an increase in TSS and total sugars of Shelly cv. Mango fruit treated with 750 g tree^− 1^.

Soil amendments enhance fruit chemical properties by improving vegetative growth and providing sugars and organic acids, reducing water stress, and ultimately enhancing fruit quality. Vermicompost and pistachio BC and 100% PWR increased WUE of eggplant^50^. Also, Shaban et al.^6^ fond increase in fruit TSS and total sugar using 14 kg BC or 100 g HD for mango trees.

## Conclusion

Biochar at rate of 14 kg per tree and hydrogel at 100 g succeeded in improving vegetative growth, nutritional status, yield and fruit quality of Zebda mango fruit. Also, biochar at rate of 14 kg was the most effective treatment which increased vegetative growth (Leaves number, leaves area, shoot length, shoot diameter) and nutritional status (N, P, K, Mg) of Zebda mango trees. Also this treatment increased fruit number by a percentage reach about 29.47 & 22.19%, fruit weight by 20.46 and 12.76%, fruit yield and water use efficiency by 55.98 and 37.79% compared to the control in the first and second seasons, respectively. Moreover biochar at rate of 14 kg per tree increased TSS by a percentage reached about 17.09 and 18.27, titratable acidity by 21.43 and 2.63%, ascorbic acid by 23.56 and 18% and total sugars by 27.92 and 3.19% compared to the control in both seasons, respectively. Biochar specially at high rate (14 kg per tree) as soil conservation and organic matter substances succeeded in improving vegetative growth, mineral content, fruiting and yield.

## Data Availability

The data generated and/or analysed during the current study are available per request to the corresponding author.
